# Adaption and pilot implementation of an autism executive functioning intervention in children’s mental health services: a mixed-methods study protocol

**DOI:** 10.1186/s40814-020-00593-2

**Published:** 2020-04-27

**Authors:** Kelsey S. Dickson, Gregory A. Aarons, Laura Gutermuth Anthony, Lauren Kenworthy, Brent R. Crandal, Katherine Williams, Lauren Brookman-Frazee

**Affiliations:** 1 San Diego State University, San Diego, CA USA; 2 Child and Adolescent Services Research Center, San Diego, CA USA; 3 Department of Psychiatry, University of California San Diego, La Jolla, CA USA; 4 University of Colorado School of Medicine, Aurora, CO USA; 5 Children’s Hospital of Colorado, Aurora, CO USA; 6 Center for Autism Spectrum Disorders, Children’s National, Washington, DC USA; 7 Rady Children’s Hospital-San Diego, San Diego, CA USA; 8 Autism Disovery Institute at Rady Children’s Hospital, San Diego, CA USA

**Keywords:** Implementation, Mental health services, Mental health, Autism spectrum disorder, Executive functioning

## Abstract

**Background:**

Youth with autism spectrum disorder (ASD) represent a growing population with significant service needs. Prominent among these needs are high rates of co-occurring psychiatric conditions that contribute to increased functional impairments and often necessitate mental health services. Executive functioning deficits are associated with ASD as well as common co-occurring conditions (e.g., attention-deficit/hyperactivity disorder) and an evidence-based intervention has been developed and tested to address executive functioning within the school context. There is an urgent need to implement indicated evidence-based interventions for youth with ASD receiving care in community mental health settings. Interventions that optimally “fit” the mental health services context as well as the complex and co-occurring mental health needs of these youth have the potential to improve key clinical outcomes for this high priority population.

**Methods:**

This mixed-methods developmental study will apply the Exploration, Preparation, Implementation, Sustainment implementation framework and a community-academic partnership approach to systematically adapt and test an evidence-based executive functioning intervention for youth with ASD for delivery in community mental health settings. Specific aims are to (1) conduct a need and context assessment to inform the systematic adaptation an executive functioning evidence-based intervention; (2) systematically adapt the clinical intervention and develop a corresponding implementation plan, together entitled “Executive Functioning for Enhancing Community-based Treatment for ASD,” (EFFECT for ASD**)**; and (3) conduct a feasibility pilot test of EFFECT for ASD in community mental health settings.

**Discussion:**

Tailoring evidence-based interventions for delivery in community-based mental health services for youth with ASD has the potential to increase quality of care and improve child outcomes. Results from the current study will serve as the foundation for large-scale hybrid implementation and effectiveness trials and a generalizable approach for different service systems of care and clinical populations.

**Trial registration:**

Clinicaltrials.gov, NCT04295512.

## Background

Autism spectrum disorder (ASD) is characterized by impairments in socialization, communication, and restricted interests and repetitive behaviors [1]. Currently estimated to affect 1 in 59 school-aged youth, individuals with ASD represent an expanding population with multiple health care and service needs [2]. Caring for autism is costly, as ASD is the youth health care condition with the highest increase in annual expenditures—projected to increase from $268 to $461 billion by 2025 [3, 4]. Youth with ASD have high rates of co-occurring psychiatric conditions (e.g., disruptive or challenging behaviors, attention-deficit/hyperactivity disorder, anxiety), estimated at 70% versus 25% for other youth [5–7], that can contribute to further functional impairments and health care needs [8–10]. Several evidence-based interventions (EBIs) have been developed, including those targeting co-occurring psychiatric symptoms. However, the majority of youth with ASD treated in the community do not have access to these EBIs. Therefore, to maximize care effectiveness and reduce long-term costs, it is critical that youth with ASD have access to indicated EBIs via their effective implementation and sustainment in routine care settings.

## Mental health services for youth with ASD

The public mental health service system plays a key role in serving school-age youth with ASD [11, 12]. Mental health providers report that youth with ASD or suspected ASD represent 21% of their caseloads [13]. Yet, the complex clinical presentations and unique challenges associated with treating youth with ASD pose significant service challenges for mental health providers. Mental health providers report frustration serving this population due to perceived limited progress and ineffective treatment strategies, and cite a need and motivation for specialized training in effective ASD interventions [13]. Caregivers of youth with ASD also report frustration and decreased satisfaction with mental health services due to providers’ limited ASD knowledge and training [14, 15]. Given the significant mental health needs of youth with ASD, targeted efforts are necessary to integrate EBIs in ways that are feasible, acceptable, and effective in regard to training providers and meeting the needs of youth with complex mental health problems.

## Existing EBIs for ASD

Despite significant efforts to develop and test EBIs targeting core ASD symptoms as well symptoms associated with co-occurring psychiatric conditions, community implementation deficits persist [16–18]. Many EBIs demonstrate poor fit with end user (i.e., therapist and client) and service settings needs, resulting in low acceptability or feasibility and ultimately contributing to limited implementation [19, 20]. For example, most mental health EBIs for ASD target specific co-occurring conditions (e.g., cognitive behavioral therapy for ASD and anxiety [21, 22]). EBIs targeting individual co-occurring disorders may pose implementation challenges, including poor fit with client clinical needs, as children with ASD presenting to community mental health services typically present with multiple co-occurring conditions [23, 24]. These mental health disorder-specific EBIs also pose a poor fit with provider training needs. Community providers face significant challenges in sufficiently mastering and delivering an EBI or, in the case of co-occurring conditions, appropriately selecting a sequence of EBIs for implementation [18, 25]. Targeting underlying clinical mechanisms that cut across co-occurring conditions and ASD represents a potentially more impactful and feasible approach to promoting community implementation through better meeting the complex clinical needs of youth served in these settings. Moreover, the increase applicability of these EBIs may also facilitate improved implementation outcomes through addressing key determinants of EBI use such as provider attitudes and perceptions of fit [26–28].

## ASD EBIs in mental health services

Until recently, most of the ASD intervention research was primarily aimed at examining the efficacy of interventions conducted in research settings or the effectiveness of delivery in non-mental health settings (e.g., early intervention, schools [21, 22, 29, 30]). Thus, there is a need to develop, test, and implement feasible ASD interventions for delivery in publicly funded mental health settings. In response to this need, Brookman-Frazee and colleagues [31] utilized a community-partnered approach to develop an intervention protocol entitled “An Individualized Mental Health Intervention for ASD” (“AIM HI”) consisting of a package of evidence-based strategies aimed to reduce challenging behaviors in youth with ASD and a corresponding therapist training protocol. AIM HI strategically targets the primary presenting problem (e.g., challenging behaviors) for youth with ASD in mental health services rather than the individual co-occurring diagnoses to more feasibly meet child clinical needs in community mental health settings. In collaboration with autism experts and community stakeholders, AIM HI was developed based on a systematic mixed-methods needs assessment of both child clinical needs and provider training needs [13, 15]. The iterative, community partnered and transdiagnostic (i.e., appropriate for a range of co-occurring psychiatric conditions) approach to develop and further refine AIM HI has been shown to increase therapist delivery of evidence-based strategies and improve child clinical outcomes [32–35]. Furthermore, AIM HI research highlights the relevance and utility of a transdiagnostic approach to improve clinical outcomes for a clinically challenging population of children with ASD receiving mental health services [36, 37]. The current study extends AIM HI research by applying the community partnered, context-based approach to target a new transdiagnostic clinical mechanism. Specifically, the current study will systematically adapt an existing EBI targeting executive functioning to optimize its fit with the mental health service system, primary end users of mental health providers, and end targets of youth with ASD. Additionally, through a corresponding implementation plan, the current project also targets implementation mechanisms of change, namely provider attitudes, perceptions of fit and intention to use, known to predict EBI use [26–28].

## Executive functioning: a potent transdiagnostic clinical mechanism

Identified as a key National Institute of Mental Health Research Domain Criteria subconstruct [38], executive functioning, or “cognitive control,” is defined as a collection of self-regulatory processes such as inhibition, planning, organizing, and flexibility necessary for goal-directed behavior [39]. Mounting evidence suggests an integral role of executive functioning in both ASD and psychiatric conditions that frequently co-occur with ASD [40–47]. Executive functioning deficits increase over the course of development and are linked and contribute to increased psychiatric symptoms in youth with ASD [45, 48–51]. Given that almost 80% of youth with ASD served in mental health services have attention-deficit/hyperactivity disorder and that executive functioning deficits underlie both attention-deficit/hyperactivity disorder and ASD, executive functioning serves as a key transdiagnostic clinical mechanism impacting psychiatric conditions in youth with ASD. Thus, interventions targeting executive functioning are highly relevant for this service setting and population. Additionally, socioeconomic status is a strong predictor of executive functioning, with youth from low-income or economically disadvantaged homes demonstrating more executive deficits [52, 53]. As youth from low-income families, including those with ASD, comprise a large portion of those served in community-based mental health clinics [54], executive deficits are extremely relevant to mental health services.

## Executive functioning as a target of EBIs

The role of executive functioning in the etiology of mental health conditions has spurred increased focus on this key construct in intervention development efforts [55–57]. A cognitive-behavioral executive functioning intervention specific to ASD, entitled Unstuck and On Target (*UOT* [58]), was developed for use in community school service settings with students with ASD. *UOT* targets impairments in flexibility, goal setting and planning, and problem solving [40, 45, 48, 59]. *UOT* is effective in improving flexibility, organization, and problem solving for youth with ASD as well as youth with attention-deficit/hyperactivity disorder when delivered in school settings [30, 60], supporting the classification of *UOT* as an EBI with good quality evidence [61]. Teachers also demonstrated high rates of fidelity, intervention satisfaction, and participation and treatment completion, supporting the acceptability and feasibility of *UOT* in community settings [30, 62, 63]. Further, trials testing *UOT* revealed no important moderators of effect in a very diverse sample (not race, language spoken in the home, income, IQ) [60, 64].

Results from *UOT* trials suggest that EBIs addressing mechanisms such as executive functioning have the potential to effectively address symptoms related to ASD as well as the range of co-occurring mental health symptoms and conditions. Additionally, findings demonstrate sustained improvements in problem solving a year after completion of *UOT*, irrespective of youth race or ethnicity and income [60]. Together, these data indicate the potential utility of *UOT* in mental health service settings. Through its high rates of feasibility and acceptability, *UOT* could help meet mental health provider training needs related to effective, targeted interventions for ASD [13]. Although *UOT* was designed and tested in school settings to be delivered by school staff, the intervention target and associated training model is potentially transferrable to mental health settings. *UOT* was designed as lesson plans specifically to be implemented in school settings and delivered in group settings by school staff. Thus, adaptation or augmentation to maximize its fit with client clinical and provider training needs and thus implementation within mental health services is needed. However, there have been no studies testing the effectiveness and utility of an executive functioning EBI in mental health settings for youth with ASD and co-occurring mental health conditions, especially one specifically developed for this service setting and high priority population.

### Current project

Executive Functioning for Enhancing Community-based Treatment for ASD (EFFECT for ASD) aims to examine the utility of *UOT* to improve key clinical and implementation outcomes for youth with ASD when systematically adapted for delivery in public mental health services. The Exploration, Preparation, Implementation, Sustainment [65] (EPIS) implementation framework will be applied to inform the systematic adaptation of *UOT* and develop a corresponding implementation plan to maximize its adoption and effectiveness in community mental health service settings. Specifically developed for public service sectors, EPIS specifies the implementation process, divided into four phases (exploration, preparation, implementation, sustainment), and implementation factors (i.e., innovation factors, bridging factors) associated with two contextual levels (inner and outer contexts). The outer context details external factors such as service environment and client population characteristics whereas the inner context refers to intra-organizational factors organizational and provider characteristics. Innovation factors detail those context-specific elements key to fit between an EBI and the context in which it is implemented. Bridging factors refers to those that span and link the inner and outer contexts. For example, system level policies, funding, partnership and collaboration, and advocacy can serve to link inner and outer contexts. Given its alignment with the focus and aims of the current project, the EPIS framework will be applied to all study activities [65, 66]. As the outer context is relatively stable, the primary focus will be on the impact of relevant inner context factors, namely provider characteristics, across the first three implementation phases (i.e., exploration, preparation, and implementation). As one of the outer/inner context “bridging factors” specified in EPIS and applied in previous ASD-focused research in mental health services [67], a community-academic partnership will also be integrated to tailor EFFECT for ASD fit and impact in mental health services settings. The proposed study has three specific aims that correspond to the first three EPIS phases:

- Aim 1. Conduct a need and context assessment to inform the systematic adaptation of *UOT* for implementation in youth mental health services.
- Aim 2. Systematically adapt *UOT* and develop a corresponding implementation plan entitled EFFECT for ASD
- Aim 3. Conduct a feasibility pilot test of EFFECT for ASD in community mental health settings.

## Targeted implementation and clinical change mechanisms

**Fig. 1:**
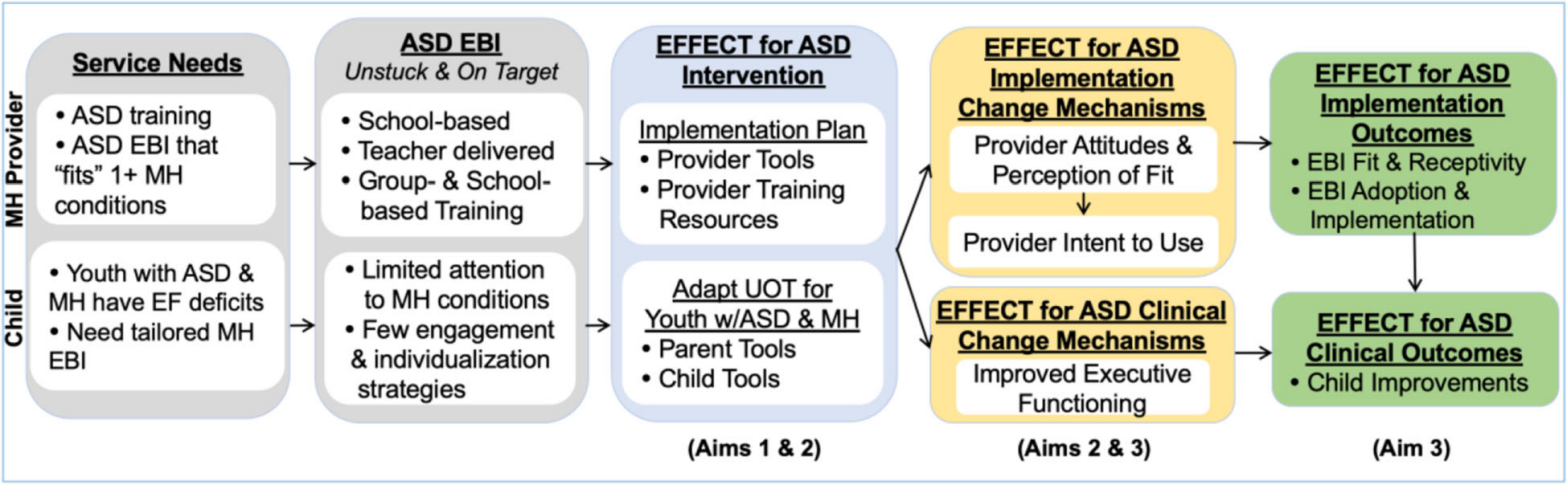
EFFECT for ASD intervention, mechanisms, and outcomes (adapted from the EPIS framework)

EFFECT for ASD will target two implementation change mechanisms affecting both implementation and clinical outcomes in mental health: (1) provider attitudes and perceptions of intervention fit and (2) provider’s intention to use (see Fig. 1). Provider attitudes towards EBIs are widely linked to specific practice behaviors and predict EBI use as well as increased fidelity following training [26–28, 65, 68]. Changes in attitudes are also known to facilitate EBI adoption and intention to use [69, 70]. Provider perceptions of innovation fit is also a critical determinant of EBI adoption and implementation in public mental health settings [71–73]. Through its transdiagnostic approach (i.e., appropriate for multiple co-occurring mental health conditions) and context-specific adaptation to maximize fit, EFFECT for ASD aims to improve provider attitudes and perceptions of EBI fit and subsequent intent and actual use through the creation of one intervention that providers perceive as being feasible, acceptable, and fitting within their practice. It also addresses their reported need for training and effective interventions [13] by allowing them to effectively serve the spectrum of co-occurring psychiatric conditions seen in youth with ASD on their caseloads. This project also targets a clinical change mechanism of improved executive functioning, a potent factor impacting both ASD and psychiatric symptoms, that can lead to significant youth improvements (e.g., reduced symptoms, reduced challenging behaviors) and care effectiveness.

## Methods

### EFFECT community-academic partnership

A community-academic partnership (CAP), defined as a partnership involving community members and academic researchers targeting a cause relevant to the community of interest [74], will be incorporated into all project aims to maximize the maximize EFFECT for ASD fit and impact in community mental health settings. In the current study, we apply a CAP model utilized in previous ASD and mental health services research [75–77]. The CAP will be comprised of key researchers and community stakeholders representing various levels of experience and roles serving youth with ASD, including mental health organization or agency leaders, mental health providers, caregivers of youth with ASD, and the principal investigator. Across all aims, the CAP will meet regularly to provide multiple perspectives and inform and guide research activities, including supporting participant recruitment, adaptation and development of EFFECT of ASD, and interpretation and dissemination of study findings and results.

### Aim 1: mixed-method context assessment to support *UOT* adaptation and EFFECT for ASD creation

A sequential mixed-method approach, with secondary quantitative methods preceding primary qualitative methods will be used to identify factors key to the adaptation of *UOT* and creation of a corresponding implementation plan for use in the mental health services context.

#### Participants

Participants (*N* = 100) will initially be recruited for the quantitative survey, with a subset of these participants (*N* = 40) also recruited to participate in subsequent qualitative focus groups. CAP members and leaders from partnered community-based mental health agencies will be asked to facilitate identification and recruitment of participants. Recruitment will occur through several methods, including in-person appearances at staff meetings, flyers posted in patient areas, and targeted email contact. Survey participants who agree to be contacted regarding future study activities will be recruited to participate in qualitative focus groups. Participants will include mental health leaders (e.g., agency leaders, executive directors), mental health providers (i.e., clinicians), and caregivers of youth with ASD either currently or previously receiving mental health services, namely psychotherapy services.

#### Procedure

Web-based surveys will be distributed using web-based software. Focus groups will occur at a centralized location identified as most convenient for participants. Separate focus groups will be conducted based on participant type in order to minimize demand characteristics and promote candid response: (1) agency leader (approximately one group), (2) mental health provider (approximately two groups), and (3) caregivers of youth (approximately two groups). Estimated time commitment and compensation for participation include 90 and 20 min and $40 and $20 for focus groups and surveys, respectively.

#### Measures

Quantitative and qualitative measures will be designed and administered to elicit information regarding inner context (i.e., within organization) targets emphasized in EPIS framework and key to the adaptation of *UOT* and development of corresponding implementation protocol. Provider and caregiver specific versions will be utilized for quantitative and qualitative measures. Table 1 lists specific survey measures. All established quantitative measures have evidence of strong reliability and validity. Measures will be adapted to assess the nature and impact of difficulties as well as knowledge and confidence with specific executive functioning constructs targeted in *UOT* (e.g., flexibility, emotional control, planning, and goal setting). Items assessing implementation outcomes (fit, feasibility, utility) will be developed or adapted to assess specific *UOT* intervention content and format. Semi-structured focus group guides with pre-selected interview questions that correspond to the quantitative surveys will be used to facilitate participant discussion of perspectives regarding: (1) the role and impact of executive functioning in mental health services, (2) executive functioning experience and training needs, and (3) key areas for adaptation of *UOT* for mental health services, including adaptations to the content, context, and training of *UOT* and associated provider training.

**Table 1:** Quantitative measures

Construct	Measure/indicator	Informant			Timeframe	
		C	Y	P	Pre/mid	Post
Aim 1 Web-based needs assessment survey measures						
Executive functioning difficulties and treatment engagement	Client engagement challenges* [78]	X		X	--	--
Executive functioning knowledge and confidence	Knowledge and confidence* [34, 78]	X		X	--	--
*UOT* intervention feasibility, utility and fit	Usefulness scale* [79]			X	--	--
Intention to use	Project developed			X	--	--
Determinants of EBI use	Multilevel EBI determinants* [80, 81]			X	--	--
Aim 2 Adaptation measures						
*UOT* adaptations	FRAME adaptations framework [82]	X		X		
CAP collaborative process	Collaborative process survey [83]	X		X		
Aim 3 Implementation change mechanism						
Provider attitudes and perception of fit	Evidence-based practice attitude scale [26]			X	X	X
Intention to use	Innovation-specific implementation intentions* [84]			X	X	X
Aim 3 Clinical change mechanism						
Improved executive functioning	NIH toolbox cognition measures [85]		X		X	X
	Behavioral rating scale of executive function [86]	X		X	X	X
	Weschler abbreviated scale of intelligence-block design [87]		X		X	X
	Executive function challenge task [88]		X		X	X
Aim 3 Implementation Outcomes						
Feasibility, acceptability, and appropriateness	Perceived characteristics of intervention scale* [89]	X		X		X
	Acceptability of intervention, feasibility of intervention, and	X		X		X
	Intervention appropriateness measure [90]	X		X		X
Implementation Process	Stages of implementation completion*			X	X	X
Uptake and intervention fidelity	*UOT* fidelity procedures [30]			X	X	X
	Provider-report of fidelity (project developed)			X	X	X
	Adaptations to evidence-based practices [78]			X	X	X
Aim 3 client outcomes						
Improved child symptomatology	Eyberg child behavior inventory [91]	X			X	X
	Pediatric symptom checklist (PSC) [92]	X		X	X	X
	Child behavior checklist (CBCL) [93]	X		X	X	X

*C* = caregiver-report; *Y* = assessments administered to youth; *P* = provider-report

*Adapted/finalized for the current project in consultation with CAP and/or mentor team

#### Data analysis

Informed by the systematic mixed-methods needs assessment of mental health services for youth with ASD in AIM HI [13], a sequential quan → QUAL mixed-methods design will be employed whereby quantitative (hence lower case “quan”) survey data will inform the development and analysis of primary qualitative focus group (hence upper case “QUAL”) [94, 95]. Consistent with current recommendations [94, 96, 97], data will be integrated and triangulated to examine convergence (e.g., do data provide similar answers to the same questions), complementarity (e.g., focus group data providing additional context to aid interpretation of survey data), and expansion (e.g., qualitative data providing explanations or new interpretations for findings produced by survey data). While the design is sequential in that quan data informs the QUAL measures, analyses will involve a bi-directional analysis and interpretation of the two data types.

### Aim 2: *UOT* adaptation and development of corresponding implementation plan to develop EFFECT for ASD

Informed by aim 1 mixed-method findings, an iterative, collaborative approach will be used to adapt and enhance the existing *UOT* intervention and create a corresponding implementation plan to maximize feasibility and fit in mental health settings.

#### Participants

Participants include CAP members, the principal investigator, and key mentors and consultants, including original *UOT* developers.

#### Procedures

Meetings will focus on providing feedback regarding aim 1 findings as well as discussion and incorporation of findings to guide adaptation of *UOT* and corresponding implementation plan development and providing feedback on drafted materials. *UOT *adaptation will include a particular focus on implementation feasibility and external validity of EFFECT for ASD models while also retaining core *UOT* principles and core components, or those key to *UOT* effectiveness. Using an iterative approach, meetings will focus on reviewing and refining drafted materials, with particular emphasis on modifications that support implementation feasibility and utility in mental health settings. The principles behind *UOT* and corresponding intervention components will be outlined, and CAP members will discuss how these might best fit into the mental health service context. Aim 1 results will be presented for validation and used for reference on key modifications. Incorporating this feedback, the principal investigator will develop the initial version of EFFECT for ASD. In subsequent meetings, CAP members will provide feedback for further revisions. This process will be used for the development and revision of all materials and *UOT* developers and mentors (Drs. Kenworthy and Anthony) will review the protocol throughout this process to assess suggested adaptations and ensure fidelity with the principles and *UOT* core elements*.*

**Fig. 2:**
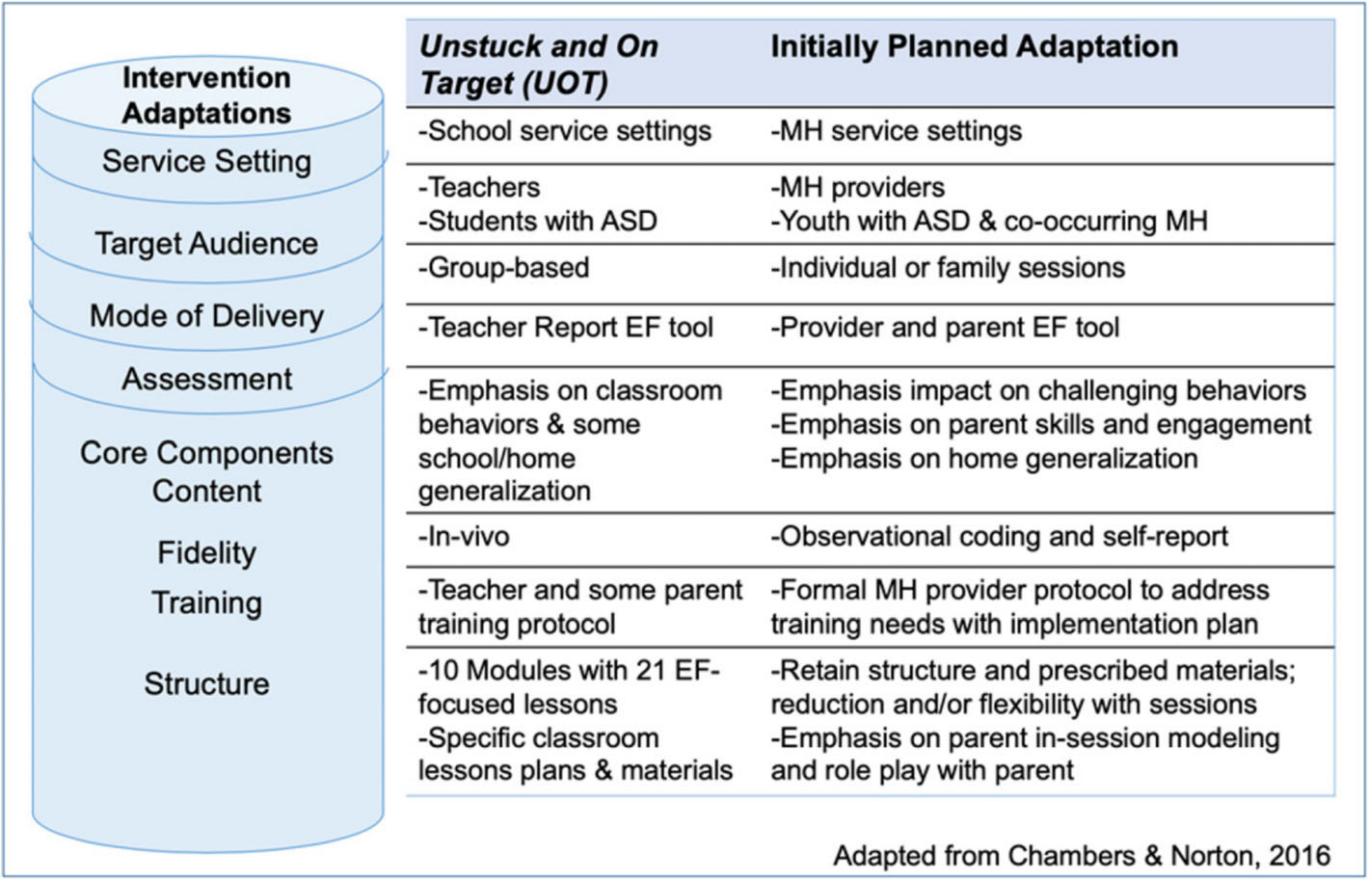
Current structure and proposed adaptations of *UOT* for EFFECT for ASD

This process will likely preserve the *UOT* components, including modules with multiple EF-focused lessons (e.g., flexibility, planning, self-monitoring, goal setting) and structured lesson plans and materials (e.g., didactic content, application activities). Possible adaptations include (1) modification of language and activities appropriately for the mental health setting (e.g., change language from “classroom” to “session,” adapt to small group or individual activities); (2) adaptation of materials and components key to mental health service context, including those targeting managing challenging behavior [83], parent engagement and skills [98], home generalization and promoting involvement of other key influencers such as other caregivers and teachers; and (3) additional content and format adaptations to better fit providers, caregiver and client needs (e.g., feedback on pattern of executive functioning deficits and appropriate supports, in-person and online tools). See Fig. 2 for a description of the *UOT* components and proposed adaptation areas informed by the Chambers and Norton’s adaptome platform [99], which systematically captures common areas of EBI adaptations to aid intervention development and implementation efforts. A corresponding implementation plan will be developed and yoked with the adapted intervention and likely include multi-method, evidence-based training [100, 101], biweekly ongoing supervision (practice with feedback), consultation, and fidelity monitoring to aid implementation and fidelity (see aim 3 the “Procedures” section). CAP meetings will occur monthly during this phase lasting from 1-2 h.

#### Measures

Quantitative measures and qualitative approaches will be utilized to characterize and document the adaptations made to *UOT* and corresponding implementation plan. Measures will be informed by the existing adaptation framework developed by Stirman and colleagues [82] and existing work applying this framework to measure and track adaptions in community-based implementation efforts [102, 103]. Specifically, the Stirman et al., framework will be used to document key aspects of the adaptation process, including how modifications were made, what was modified, at what level of delivery modifications were made, and the type or nature of modifications. See Table 1 for list of aim 2 measures.

### Aim 3: feasibility pilot test of EFFECT for ASD in mental health settings

**Fig. 3:**
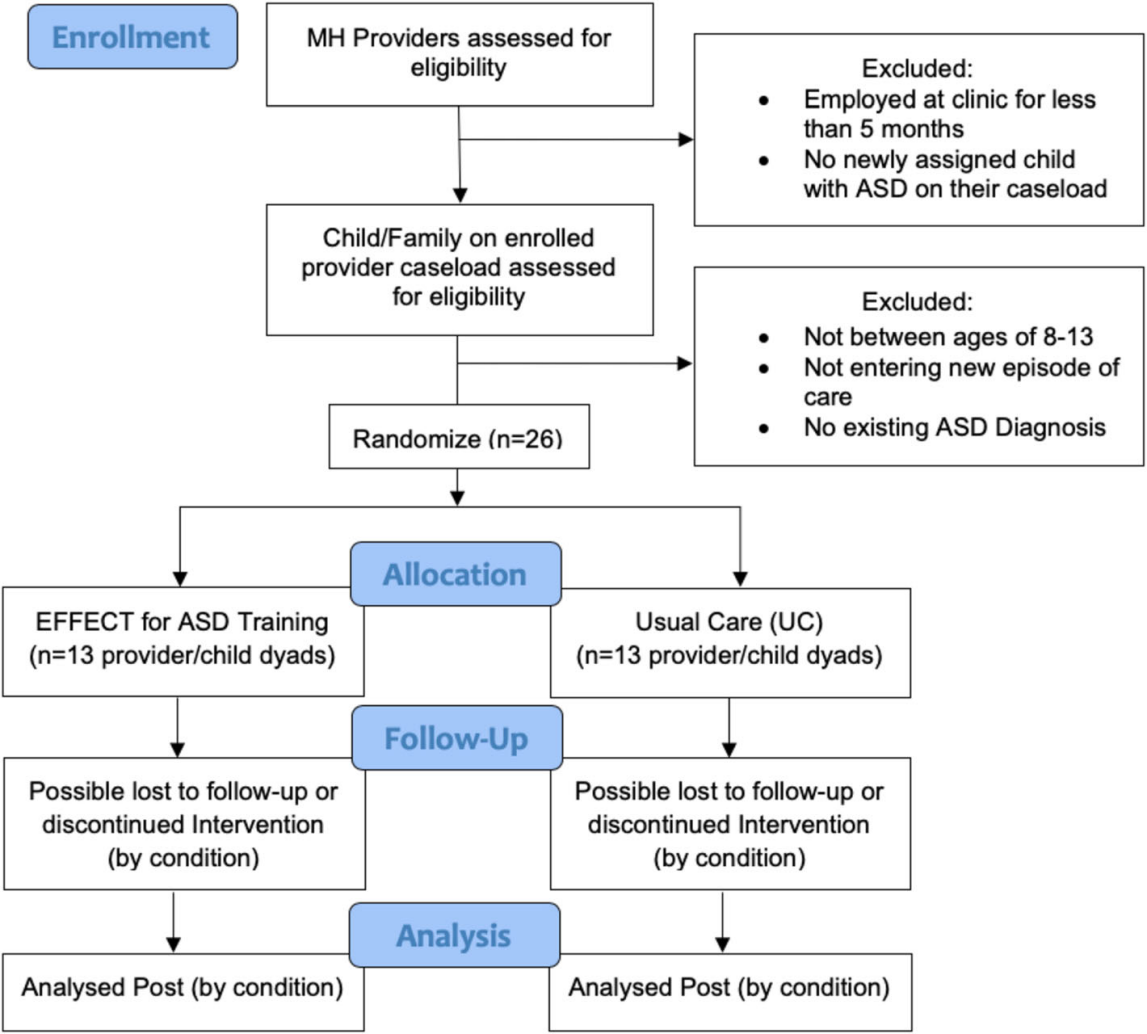
Aim 3 EFFECT for ASD consort diagram

The primary goal of this aim is to conduct a pilot test examining the feasibility of EFFECT for ASD in mental health settings. Pilot study will evaluate (1) feasibility, utility, and perceived fit of EFFECT for ASD in publicly funded mental health service settings; (2) EFFECT use and adherence; and (3) preliminary youth clinical outcomes, including changes in executive functioning and challenging behaviors (see Fig. 3).

#### Participants

Provider-youth dyads (*N* = 26) will be recruited from partnered community-based mental health clinics. Sample size was informed by prior feasibility pilot studies focused on community-based service interventions for youth and recommendations regarding appropriate statistical analyses [34, 104, 105]. Eligible providers include those employed at one of the three participating clinics for at least 5 months, and have a child with ASD newly assigned to their caseload. Children and their families will be eligible if the child is between the ages of 8-13, has an existing ASD diagnosis according to case records, and are entering a new episode of outpatient mental health care. Facilitated by CAP members, partnered organizational leaders, and eligible providers will be recruited via similar methods as aim 1. Recruitment will occur through several methods, including in-person appearances at staff meetings, flyers posted in patient areas, and targeted email contact. Aim 1 provider participants who consented to be contacted regarding future studies will also be recruited. Youth/family recruitment will be linked to provider recruitment, with interested or enrolled therapists supporting identification and recruitment of eligible youth/family participants through obtaining permission to be contacted by the research team. The research team will subsequently consent and enroll families, resulting in 26 provider-child dyads.

#### Procedures

All participants will be provided information about the study from research staff and provide consent obtained prior to data collection. Enrolled provider-youth dyads will be randomly assigned to one of two conditions: EFFECT for ASD training and waitlist control. Providers will be randomized by an independent researcher via computer-generated randomization to one of the two study conditions per study (with group assignment based on even versus odd digits). Providers in the treatment condition will receive training according to the protocol developed in aim 2 whereas providers in the waitlist condition will receive training approximately 6 months after those in the treatment condition. Participants will be asked to complete measures pre (baseline) and six-months post implementation of EFFECT for ASD or at the beginning and end of 6 months for the control condition. Across conditions, provider participants will submit video recordings of intervention sessions with enrolled families and a corresponding provider-report fidelity measure to assess intervention fidelity. As with aim 1 findings, CAP will aid with interpretation of findings.

#### Measures

Quantitative measures and qualitative approaches will be administered to provide a comprehensive understanding of the primary implementation and secondary clinical outcomes of EFFECT for ASD, including feasibility, acceptability, uptake, fidelity, and effectiveness of EFFECT for ASD. Quantitative youth outcome measures will be administered pre and post, whereas implementation outcomes will be collected at post (see Table 1). Fidelity measures which will be assessed throughout EFFECT for ASD implementation. Additional objective metrics of implementation outcomes related to the research process (e.g., enrollment/withdrawal rate, data collection completion rate) will also be examined given prior data highlighting the influence of perceptions regarding research and data collection activities on implementation [67]. For the qualitative focus interviews, semi-structured interview guides with pre-selected questions will be used to gather participant perspectives pertaining to EFFECT for ASD feasibility, acceptability, appropriateness, and effectiveness. As with aim 1, respondent-specific (e.g., provider, caregiver, youth) versions will be administered. See Table 1 for list of all aim 3 measures.

#### Analyses

Descriptive analyses and examination of qualitative themes related to perceptions of the feasibility, acceptability, and appropriateness of EFFECT for ASD will be used to evaluate key implementation outcomes. For quantitative measures, criteria for determining sufficient feasibility, acceptability, and appropriateness will be applied, including examination of mean-level ratings on relevant measures (see Table 1) and whether mean scores are greater than or equal to ratings indicating perceived feasibility, acceptability, and/or appropriateness (e.g., ≥ 4 on feasibility and acceptability of interventions measures, ≥ 4 on subscales on the perceived characteristics of intervention scale). Scores will also be compared to prior relevant literature. For example, fidelity scores will also be analyzed and compared to prior trials of *UOT* to determine if providers achieve similar fidelity levels, indicating appropriate uptake and fidelity. Finally and informed by current recommendations and prior work [106], objective measures of outcomes (e.g., recruitment and enrollment, proportion of sessions completed, attrition) will also be examined. Intervention effects analyses will also be conducted, including group (intervention vs. control) × time (pre to post), will be conducted to examine changes in outcome measures over time; analyses will be conducted using recommended procedures (e.g., maximum likelihood) to adjust for missing data and non-normality of outcome variables. Informed by similar pilot feasibility trials, the targeted sample size (*N* = 26) allows for dropout rates similar to that of prior pilot studies in the same setting while also meeting the threshold for sufficiently precise parameter estimates [34, 104, 105, 107]. Per current recommendations [108], direction of effects and effect size estimation will be examined given the small sample size. Consistent with a type III scale-out [109], this pilot study adapts an evidence-based intervention for a new population and delivery systems and will borrow statistical strength by comparing findings to prior studies regarding evidence of *UOT.* As such, we anticipate an effect size that approximates the medium effect observed in the *UOT* randomized-clinical trial [30] (average Cohen’s *d* = .55). Qualitative and mixed-method analyses of structured interviews will utilize the same procedures as in aim 1.

## Discussion

Youth with ASD are a high priority population with significant service needs and challenges, and ASD EBI implementation is limited in community settings. Improving receipt and efficacy of community mental health services will have a positive public health impact through reduction in health care needs and costs. This study aims to improve the effectiveness of community-based services through the development and implementation of a feasible, transdiagnostic intervention targeting potent mechanisms of action underlying ASD and an array of co-occurring mental health problems.

The current project builds directly on prior implementation research within the mental health service context, including those specifically targeting youth with ASD and encompasses several important innovations that will expand the field of implementation science. Specifically, this work expands upon and complements the transdiagnostic approach of AIM HI through the selection of an EBI targeting key transdiagnostic clinical mechanisms that can be used across profiles of mental health disorders within the context of ASD. This serves to increase EBI applicability and coverage for the multiple co-occurring psychiatric conditions in youth with ASD receiving mental health care and address provider-reported training needs while minimizing needed time and resources for training. Also complementary to the development and refinement of AIM HI, this project utilizes a community-partnered approach to systematically adapt an existing EBI and develop an implementation plan within the context of the EPIS framework and a CAP. This approach facilitates the incorporation of inner context factors and intimate, local knowledge critical to maximizing its “fit” with end user and within natural context constraints. This approach of designing and adapting interventions for end-users results in improved clinical and practice outcomes through capitalizing on an existing effectiveness of the intervention while optimizing fit and feasibility and thereby accelerating the implementation and receipt of effective care for this high priority and traditionally challenging population.

Finally, the current study aims to simultaneously test the implementation of EFFECT for ASD in mental health settings and gather pilot data on clinical effectiveness (youth outcomes). This hybrid effectiveness-implementation design [110] builds and expands upon previous UOT effectiveness data and collects data on (1) implementation mechanisms (provider attitudes and intent to use) related to implementation outcomes and (2) clinical mechanisms (changes in executive functioning) related clinical outcomes. Through its focus on key mechanisms of change as well as an EBI that specifically targets a key mechanism consistent with research domain criteria [38], it is responsive to urgent calls and prioritization for further articulating and evaluating mechanisms underlying desired clinical and implementation outcomes [38, 111, 112]. Additionally, this project extends proven techniques to address a new target of treatment (executive functioning) that drives major health, mental health, vocational, and even legal outcomes [113]. Together, these innovations have a strong likelihood of impacting multiple implementation and clinical outcomes, including increased provider EBI adoption and use and improved care effectiveness for youth with ASD, a complex, high priority clinical population.

### Next steps

Once developed and pilot tested, findings will inform the development of a larger-scale effectiveness-implementation hybrid type 2 trial of EFFECT for ASD with simultaneous examining EFFECT for ASD clinical effectiveness and implementation plan or strategies. Our primary feasibility and acceptability outcomes will support assessment of readiness for a large-scale trial. Additionally, we are leveraging the focus on scaling out an EBI with good effectiveness and implementation outcomes as well as the use of established approaches known to facilitate the successful development of an effective and feasible intervention in children’s mental health settings to support these next steps. Further large-scale studies will allow for evaluation within the context of broader, more varied mental health organizations or systems providing mental health care to youth with ASD, including evaluation of further multilevel (e.g., system, leader, provider characteristics) factors on EFFECT for ASD adoption and use. Additionally, future studies will also permit examination of methods to optimize EFFECT for ASD, including identification of the smallest effective dose and/or active ingredients of *UOT* for youth. Finally, the current project is focused specifically on adapting and testing an executive functioning intervention for youth with ASD and co-occurring mental health conditions. However, it is an entry point for generalizable application of EFFECT for ASD to other clinical populations and service settings where executive functioning significantly impacts community-based care for youth. This will help provide community mental health providers with the training they require to address the growing service demands of youth with complex clinical presentations such as youth with ASD and co-occurring mental health conditions.

### Trial status

The current project has received Institutional Review Boards approval from San Diego State University as well as partnered health care organizations and broader County Department of Behavioral Health Services. At time of submission, aim 1 quantitative survey data collection has begun.

## Data Availability

The application described in this manuscript is freely available. Please contact the lead author for more information.

## Abbreviations

AIM HI: An Individualized Mental Health Intervention for ASD
ASD: Autism spectrum disorder
CAP: Community-academic partnership
EBIs: Evidence-based interventions
EFFECT for ASD: Executive functioning for enhancing community-based treatment for ASD
EPIS: Exploration, preparation, implementation, sustainment framework
*UOT*: Unstuck and On Target
